# Guidance for reconciling patent rights and disclosure of findings at scientific meetings

**DOI:** 10.1186/1478-4505-8-15

**Published:** 2010-05-25

**Authors:** Nathaniel B Lipkus, Jocelyn E Mackie, Peter A Singer

**Affiliations:** 1 Gilbert's LLP, 49 Wellington Street East, Toronto, Ontario, M5E 1C9, Canada; 2 McLaughlin-Rotman Centre for Global Health, University Health Network and University of Toronto, 101 College Street, Suite 406, Toronto, Ontario, M5G 1L7, Canada

## Abstract

Open collaboration and sharing of information among scientists at scientific meetings can foster innovation and discovery. However, such sharing can be at odds with potential patenting and commercialization objectives. This tension may be mitigated if certain procedures are followed in the context of scientific meetings. The article first discusses what makes a scientific finding patentable and then sets out four specific patent issues for scientists to consider before attending a scientific meeting and sharing their research. Finally, it provides recommendations on how scientists can best protect their intellectual property rights while sharing information at scientific meetings.

## Review

Scientific communities thrive on collaboration, which requires sharing information. Once discovered and disclosed, however, valuable information is forever in the public domain. Information is thus the classic example of a public good: once made available information can be possessed by any number of individuals at once, and becomes impossible to control [1]. Without some form of reward, there may be insufficient incentive to encourage investments that yield socially valuable information [2].

One way by which society rewards socially useful ingenuity is through patents, which are exclusive rights to the use of an invention. Exclusivity protection for inventions has been recognized for centuries [3], and a corporation's patent portfolio may be its most valuable asset. Academic institutions have also come to increasingly value and apply for patents.

At the intersection of academia and the patent system is an inevitable tension between the imperative of information dissemination and the pursuit of profit [4]. This tension is, in part, a direct consequence of a universal requirement for patent protection - namely, that a patent will not issue, or will be declared invalid, if the claimed invention was previously disclosed to the public. The risk of forfeiting rights to a patent through careless disclosure can lead scientists to purposely hide their discoveries to preserve their economic rights. One study, by Grushcow, found that scientists who eventually patent their work appear to withhold presentation of their data at scientific conferences for periods of months, or even years, and that academic and nationally funded scientists who were *not *seeking a patent presented their abstracts over a year earlier than scientists who were seeking patents [5]. Another study found that secrecy is on the rise among academic researchers (particularly those in the life sciences), due to a desire to participate in patenting and technology transfer [6].

The relationship between patent protection and disclosure of research findings is complex, and in some circumstances, patents may speed disclosure. For example, the patent system does promote eventual disclosure soon after a patent is filed, whereas the absence of adequate patent protection could promote long-term secrecy of inventions so that they may be protected as trade secrets [7]. Further, where multiple parties are racing for patent protection, the lagging party may choose to publish their findings prematurely to limit the availability of a patent for the leading party [8].

Nevertheless, the overarching tension between protection and dissemination of research findings may affect global scientific collaborations, even those with a humanitarian objective like the "Grand Challenges in Global Health" initiative. Each year, 44 research teams with members from 33 countries assemble to discuss their findings. At one of these meetings, a scientist declined to provide specific details about the findings of a particular aspect of his research. The reason given was a concern about prematurely disclosing information that could threaten the granting of a patent.

Much discussion and debate ensued among the meeting participants as to the validity of the scientist's decision not to disclose. Was the scientist undermining the collaborative nature of the initiative? Or was his position a prudent one that would be more likely to ensure the discovery reached those who needed it in the developing world? While previous articles have examined academia-industry patenting with respect to publications and conferences [1,6,9-11] there has been little explication of how these factors influence scientific collaboration and collaborative scientific meetings. The purpose of this article therefore is to provide researchers and research funders with analysis and recommendations of patenting issues related to scientific collaboration and meetings.

## What Makes a Scientific Finding Patentable?

Patentability is not typically a function of how much work is done to arrive at a result; rather, it lies in the unexpectedness or difficulty of an invention and the reduction of that invention to a commercially useful result [12]. There are three basic requirements for patentability of an invention: novelty, non-obviousness, and utility (see Appendix 1) [13-15]. Novelty pertains to the timeliness of the disclosure of the scientific finding. A patent may be available only as long as there is no "prior art" (document, product, presentation, etc.) that has already disclosed each element of the invention [16].

Non-obviousness, or the requirement of an "inventive step", imposes a threshold of ingenuity to the scientific finding. Even if the subject matter has never been publicly disclosed, if it would be obvious for an ordinary person skilled in the relevant field based on the literature and common body of knowledge in that field, then the subject matter is not deserving of a patent [17-19].

Utility means the invention disclosed in the patent must 'work'. Society will not reward a useless finding, even if it is new and not obvious. For example, a new drug delivery system that fails to make an active ingredient available in the body does not attract a patent. Only once someone finds a use for the drug delivery system might a patent be available.

Finally, some subject matter may not be patentable, such as scientific principles and abstract theorems - e.g., the laws of physics. In some places, methods of medical treatment - such as surgical methods or dosage regimens - are not patentable. Genetically-modified living beings, genes in isolation, or even an oncogenic mouse are patentable in some jurisdictions, and to varying extents [20].

## Attending a Scientific Meeting? Specific Patent Issues to Consider

### 1. Timing of Disclosure

Ordinarily, in considering whether to patent, inventors will want protection in at least two markets - the United States and Europe - and then often Japan also. The desire for protection elsewhere will depend on the commercial strategy of the inventor or their financial sponsor. Before disclosing their invention, a potential patent applicant should consult with a patent attorney to craft a patent strategy and learn what conduct could jeopardize that strategy in their markets of interest. For inventors at academic institutions, this service may be available through a university technology transfer office.

The patent system in some jurisdictions affords leeway to patentees to disclose their scientific findings before applying for a patent. However, that leeway is neither universal nor everlasting. In the United States, inventors have one year from the time they first disclose their scientific findings to apply for a patent for their work. This one-year grace period is helpful, and promotes disclosure to some extent, but can be risky. Only disclosure made by the inventor - not necessarily members of their team - during this one year is immunized. Furthermore, in Europe, the inventor does not benefit from any such grace period so if patent protection is sought in Europe, secrecy as to the content of the invention is essential until the European patent application has been filed.

### 2. Extent of Disclosure

The amount of information that a scientist discloses publicly has implications on the patentability of her findings, as well as those of other scientists in the field.

The potential risks associated with disclosure in a confidential setting will vary according to the ease of the invention's replication. Some inventions are easily replicable once disclosed - such as the identification of a key receptor in the body. Other inventions may only be replicated over several years - such as the findings from a long-term study carried out in monkeys. In determining whether to disclose findings in a scientific meeting, researchers should assess how realistic it is for a competitor to copy the disclosed findings before the researcher is able to file for patent protection.

In the US, to offset the risk of getting "scooped" by rivals, inventors can file provisional patent applications that act as placeholders for a more careful definition of the property claimed by the inventors. This affords the inventor latitude in disclosing such findings in other forums.

Once a patent application has been filed, the calculus governing the extent of disclosure changes. Whereas disclosure prior to a patent application risks undermining an eventual patent, disclosure after the application is filed will not compromise that application. On the contrary, extensive disclosure after filing a patent application may bar future patent applications in the field - both by the discloser and by competitors. Therefore, after a patent application is filed, the commercial interests of the inventor may align with the scientist's interests in disclosure - say as much as possible about the research findings, not only to advance academic discourse but also to thwart competitive patenting opportunities.

### 3. The Audience

Whether any given disclosure has been made "public" is a matter of evidence and depends on the circumstances of the disclosure. A document published in a peer-reviewed journal commonly circulated within the relevant community is of course public, but other disclosures may not be.

A fundamental aspect of patent law is that an invention is not novel if it has ever been disclosed in the past (with some exceptions which are beyond the scope of this paper) [21,22]. Therefore, even an article in an obscure journal would appear to preclude subsequent patentability, as long as it taught each of the key features of the invention and contains enough information to enable a fellow scientist to practice the invention. Where a past article does not contain the entirety of the invention, a patent for the invention may only be invalidated on the basis of the obviousness doctrine.

A poster presentation or casual conversation raises the question of oral disclosures. For US patentability purposes, only an oral disclosure that takes place in the US will factor into the patentability decision for a US patent. This is a particularly important consideration for groups of international scientists who could choose to have meetings outside of the US. For Europe, however, an oral disclosure anywhere in the world is considered.

If there is a duty of confidentiality between the parties or if a contract existed either written or oral stipulating that the findings would be kept confidential, the disclosure may legally be considered a trade secret and therefore not qualify as publication. In a controlled setting, a non-disclosure agreement can be a productive way of sharing information without causing that information to be deemed "public" for the purposes of patentability. Such an agreement would set out the designation of the shared information as secret and the obligation of the recipient to keep the information secret. One caveat, though, is that if a counterparty to a non-disclosure agreement discloses the invention in violation of the agreement before the inventor has filed for patent protection, patentability may be lost [23]. The only remedy will be a damages claim for breach of the confidentiality agreement.

In practice, even the most sophisticated companies often rely on non-disclosure agreements to explore the commercial viability of their inventions even before applying for patents. This is normally seen as a safe way for companies to capitalize on their inventions where they are unable to fully develop their inventions alone. Most companies fortunate enough to be confidentially exposed to new technologies will respect the confidential arrangement in order to demonstrate their reputation for being respectful of rights among companies in their industry.

In the scientific meeting context, a non-disclosure agreement could have a similar disciplining effect among researchers as it does among companies in large industries. Scientists seen as "scooping" inventions of other attendees in violation of non-disclosure agreements would develop poor reputations among all attendees of a meeting, and would consequently be seen as untrustworthy within the academy. Non-disclosure may therefore provide the appropriate degree of discipline at the outset of scientific meetings to ensure mutual respect for the rights of researchers at the meeting.

Finally, the circumstances of any disclosure are important. If two researchers are employees of a larger organization, they may be free to share their findings within the organization only. On the other hand, disclosure during a conversation between representatives of two market competitors may well lead to the disclosure being used against the patent applicant should its competitor seek to undermine the patent. Less clear is the situation where findings are disclosed between two members of a scientific team, funded by the same grant but in different universities. As mentioned above, a well-drafted non-disclosure agreement may render the contents of either of the latter two conversations non-public.

### 4. The Irrelevance of Why

The social importance of an invention neither renders it more or less patentable, for the most part. The patent system is completely agnostic to the field of the invention or its potential social benefit - the same rules apply to AIDS medicines as to gardening equipment.

There is one caveat, however, of particular relevance for doctors. As a matter of public policy, some countries have refused to permit patents for subject matter normally within the realm of the professional skill of doctors. These "methods of medical treatment" may include such things as surgical techniques and dosage regimens. The World Trade Organization has permitted countries to provide exceptions to patentability for such subject matter; Europe and Canada have availed themselves of such an exception, and the United States has not.

The overall inflexibility of the patent system to adapt to different contexts places the onus on scientific communities to adapt to the realities brought about by patents. Collaboration between the public and private sectors and interests is critical to optimizing information-sharing among those with the power to solve difficult problems.

For example, the Bill & Melinda Gates Foundation developed a Global Access Strategy for its grants, which aims to ensure that any global health solutions that arise from the funded projects will be made accessible to people most in need within developing countries and that knowledge gained through discovery is promptly and broadly made available to the scientific community. Scientists are required to describe how potentially patentable information will be handled. While the scientist (or their institution) retains the intellectual property, many of the agreements used in the initiative provide 'march in rights' to a non-profit or academic institution if the commercializing party fails to live up to the global access commitments. This approach is an example of how organizations and scientists can establish guidelines to promote dissemination and sharing of information while still recognizing the realities of the patent system and related commercial objectives.

## Guidance for Researchers and Funders

We offer ten suggestions for retaining patent rights without unnecessarily withholding information in scientific meetings (see Appendix 2):

## Before the Meeting

### 1. Clarify who owns the intellectual property flowing from collaborative research

The reality of today's research collaboration is that results from research funded by private interests often do make their way into scientific meetings. Participants should not be made to guess as to who will own the rights flowing from their work. A clear ownership policy from the start on the part of the funding organization (or organizations) is essential [24].

### 2. Use non-disclosure agreements in scientific meetings but only as needed

Participants can contractually bind themselves to keep the research findings of fellow researchers confidential. Any agreements should cover only those meetings where sensitive information is being exchanged and a level of protection is required. It must be recognized, though, that confidentiality agreements can chill valuable collaboration by silencing contracting parties, and so they should not be overused or made too onerous. In most cases, it would be overbroad to include traditionally public meetings, such as annual meetings of not-for-profit organizations, within the scope of a confidentiality agreement. Penalties for breaching confidentiality agreements should be no more than is necessary to adequately deter disclosure.

### 3. Consider holding meetings outside of the US due to US patent law

Currently, only oral disclosures made in the US are taken into consideration in evaluating US patentability. Unless and until this situation changes, oral disclosures at foreign meetings should not forfeit US patent rights. Be cautious when relying on this strategy, as it is specific to the US, and US law could change.

## At the Meeting

### 4. Be wary of meetings that include all experts in a field

Efforts to maintain confidentiality with a non-disclosure agreement or other agreement may be pointless if all the members of a field are present. The information may no longer be considered a secret and it is possible that a court would consider disclosure in such consequences to be a public disclosure that invalidates a later-filed patent.

### 5. Do not publish detailed meeting minutes

If contents of a meeting are written down and published, that disclosure will count against the discloser by patent offices examining the novelty of the discloser's subsequent patent application. The less detailed the meeting minutes, the less likely such minutes will contain a detailed enough description of the findings to undermine patentability.

### 6. Provide a schedule for eventual disclosure if disclosure at a meeting is not possible

Delays in filing for patent protection are often not the fault of the researcher, and researchers should - as a courtesy to fellow collaborators - ensure that if they are unable to share information on a particular meeting date, that they can provide some indication of when disclosure will be made available. Since disclosure will presumably be extensive within the patent application's specification, circulation of the patent application itself to fellow researchers on a date committed to in advance (under a non-disclosure agreement, if desired) can alleviate a past refusal to disclose for patentability purposes.

### 7. Talk to a patent attorney, or a technology transfer office

An independent attorney with knowledge of the patent system can help an inventor understand the implications of disclosure in the context of his own invention. Where available, technology transfer offices at universities can also act as a bridge between the inventor and the patent system, and even participate in the funding of the invention's commercialization.

### 8. Consider filing early for patent protection

Early patent filing can provide tremendous benefits. First, once an application has been filed, the content of subsequent disclosures will not forfeit patent rights. Second, if other researchers are working toward similar solutions, filing a patent application secures first-in-time rights to the invention for the applicant. In countries other than the United States, this early filing is dispositive of any dispute over ownership. In the US, it minimizes the costs associated with obtaining a patent at the end of the day, since less earlier-published literature will be available to interfere with the patent application.

### 9. Before filing a patent application, be cautious in disclosing research results

Absent a non-disclosure agreement in a scientific meeting, disclosure should be confined to information already in the public domain. Scientists may also disclose the problem they are interested in solving, however even that disclosure may evidence a motivation to solve a problem in a certain way. If patent protection is desired, scientists should be mindful that disclosure of findings before filing may compromise patentability. An early patent filing policy can render this concern moot.

### 10. After filing a patent application, consider disclosing extensively as part of a collaboration-friendly commercial strategy

Once the patent application has been filed, there may be no further interest in keeping research findings secret. In fact, more extensive disclosure of research findings can sometimes bolster a researcher's commercial interests. Broad disclosure can forestall competitors by creating a commons of information even broader than the boundaries of the patent. This makes it much harder for others to patent inventions in the area, and allows society to benefit from the follow-on research deriving from the invention without paying monopoly prices. However, where the inventor is considering further incremental inventions in the field, extensive disclosure could make patenting such potential inventions more difficult.

## Conclusions

This article has attempted to provide background information to assist researchers in understanding what patents are about, while also offering strategies for reconciling competing interests in collaborative scientific meetings.

This article was intended to provide guidance within the context of existing patent laws. However, legislative reform creating exemptions for oral disclosures or instituting grace periods for certain socially-beneficial oral disclosures are avenues that would reduce the risks of disclosure for scientists and funders. For example, Grushcow suggests amending the US *Patent Act *to specifically allow for early data sharing at academic conferences without threatening patentability [5]. Bagley suggests creating an opt-in system in the US where academic researchers could choose to have two years instead of one to disclose the invention before filing a patent application [6]. Grushcow and Bagley, however, recognize that such amendments have their failings. These amendments would not protect a scientist from the laws in other countries (unless harmonization occurs), they will not assist industry sponsored research, and they appear to contradict the policy behind the disclosure rule - i.e. a reluctance to allow inventions already in the public domain to be reclaimed by the inventor [5].

Until such changes to the patent laws are adopted the recommendations provided above should help guide discussions between researchers and funders. It is our hope that these guidelines can help calibrate disclosure policies, agreements and perhaps eventually a model declaration so that academic and commercial interests are preserved in the context of vital collaborative research.

## Appendix 1: What makes a Scientific Finding Patentable?

1. Is the invention new?

2. Is the invention novel or non-obvious?

3. Will the invention work, as described?

4. Is the subject matter patentable?

## Appendix 2: How to best protect your intellectual property rights while sharing results at scientific meetings

### Before the Meeting

1. Clarify who owns the IP flowing from collaborative research.

2. Use balanced non-disclosure agreements in scientific meetings but only as needed.

3. Consider holding meetings outside of the US due to US patent law.

### At the Meeting

4. Be wary of meetings that include all experts in field.

5. Do not publish detailed meeting minutes.

6. Provide a schedule for eventual disclosure, if disclosure at a meeting is not possible.

### Timing Your Disclosures

7. Talk to a patent attorney, or a technology transfer office.

8. Consider filing early for patent protection.

9. Before filing a patent application, be cautious in disclosing results.

10. After filing a patent application, consider disclosing extensively as part of a collaboration-friendly commercialization strategy.

## Competing interests

The authors declare that they have no competing interests.

## Authors' contributions

PAS conceptualized the idea and NBL and JEM provided critical legal content; all authors participated throughout the drafting process and approved the final manuscript.
